# Clinical Evidence for Three Distinct Gastric Cancer Subtypes: Time for a New Approach

**DOI:** 10.1371/journal.pone.0078544

**Published:** 2013-11-12

**Authors:** Alessandro Bittoni, Mario Scartozzi, Riccardo Giampieri, Luca Faloppi, Maristella Bianconi, Alessandra Mandolesi, Michela Del Prete, Mirco Pistelli, Luca Cecchini, Italo Bearzi, Stefano Cascinu

**Affiliations:** 1 Clinica di Oncologia Medica, AOU Ospedali Riuniti-Università Politecnica delle Marche, Ancona, Italy; 2 Scuola di Specializzazione in Oncologia Medica, Università Politecnica delle Marche, Ancona, Italy; 3 Anatomia Patologica, AOU Ospedali Riuniti-Università Politecnica delle Marche, Ancona, Italy; Pontificia Universidad Catolica de Chile, Faculty of Medicine, Chile

## Abstract

**Background:**

Recently, a new classification for gastric cancer (GC) has been proposed, based on Lauren's histology and on anatomic tumour location, identifying three subtypes of disease: type 1 (proximal non diffuse GC), type 2 (diffuse GC) and type 3 (distal non diffuse GC). Aim of our analysis was to compare clinical outcome according to different GC subtypes (1,2,3) in metastatic GC patients receiving first-line chemotherapy.

**Patients and Methods:**

Advanced GC pts treated with a first-line combination chemotherapy were included in our analysis. Pts were divided in three subgroups (type 1, type 2 and type 3) as previously defined.

**Results:**

A total of 248 advanced GC pts were included: 45.2% belonged to type 2, 43.6% to type 3 and 11.2% to type 1. Patients received a fluoropyrimidine-based chemotherapy doublet or three drugs regimens including a platinum derivate and a fluoropyrimidine with the addition of an anthracycline, a taxane or mytomicin C. RR was higher in type 1 pts (RR = 46.1%) and type 3 (34,3%) compared to type 2 (20,4%), (p = 0.015). Type 2 presented a shorter PFS, median PFS = 4.2 months, compared to type 1, mPFS = 7.2 months, and type 3, mPFS = 5.9 months (p = 0.011) and also a shorter OS (p = 0.022).

**Conclusions:**

Our analysis suggests that GC subtypes may be important predictors of benefit from chemotherapy in advanced GC patients. Future clinical trials should take in account these differences for a better stratification of patients.

## Background

Despite its incidence in Europe and North America has declined over the last three decades, gastric cancer (GC) is still the second cause of cancer-related deaths worldwide [1], representing a challenging problem for oncologists. Although different histological subtypes of GC have been identified, in the daily clinical practice and for the purpose of medical management, GC is usually considered as a single disease.

The World Health Organization and Lauren's classification system have described two main histological types of gastric cancer, intestinal and diffuse subtype, representing two entirely different epidemiological and pathological entities. The intestinal-type, characterized by cohesive neoplastic cells organized in gland-like tubular structures, is more common in men and older people; it usually arises following chronic infection by *H. Pylori* with consequent chronic inflammation and atrophic gastritis [2], [3]. On the other hand, diffuse GC, histologically characterized by infiltration and thickening of the stomach wall, occurs more commonly in women and young patients; it is usually independent from inflammation processes and can be hereditary, as a result of germline mutation of E-cadherin [4]. The gastro-esophageal junction (GEJ) cancer, arising from proximal stomach, is currently considered as a third distinct entity. Despite the overall decrease in GC incidence, the incidence of GEJ adenocarcinoma in Western countries has increased over the last decades. Gastroesophageal reflux disease and obesity are considered the major risk factors for GEJ cancer and Barrett esophagus represents a precancerous lesion for this type of tumour (Table 1).

**Table 1:** Gastric cancer subtypes features.

Gastric Cancer Subtype	Prevalent Risk Factor	
**Proximal, Non-Diffuse GC (type 1)**	*Environmental*	Tobacco use, Alcool
	*Clinical*	Obesity, High BMI, GERD
	*Genetic*	Non specifically identified
**Diffuse GC (type 2)**	*Environmental*	Non specifically identified
	*Clinical*	unknown
	*Genetic*	CDH1 mutation, Family history (non CDH1 mutant)
**Distal, Non-Diffuse GC (type 3)**	*Environmental*	High dietary salt, Tobacco, Age (peak 50–70 yrs), Low Fruit/vegetables intake
	*Clinical*	*H.Pylori* infection, Use of NSAIDs
	*Genetic*	Immune regulatory SNPs

*Modified from Shah et al* [*8*].

BMI: Body Mass Index, GERD: Gastroesphageal Reflux Disease, CDH1: Cadherin-1,

NSAIDs: Non-Steroideal Anti-Inflammatry Drugs, SNPs: Single Nucleotide Polymorphism.

This classification is also supported by clinical differences between the GC subtypes. In particular, it is well known that intestinal and diffuse GC have different metastatic pattern, with diffuse type more likely to spread to the peritoneum compared to intestinal GC. [5]. It has also been demonstrated that patients with resected diffuse GC have a worse clinical outcome, when compared stage-by-stage, to intestinal GC patients [6].

Recently, these epidemiological, pathological and clinical data have been incorporated to define a new classification of GC that identifies three tumour subtypes: type 1, proximal non-diffuse GC, with the bulk of the tumour (>80%) located in the gastric cardia and characterized by a non-diffuse pattern of infiltration; type 2, diffuse GC, located anywhere in the stomach with an entirely diffuse pattern of infiltration; type 3, distal non-diffuse GC, with the bulk of the tumour located in the distal or mid stomach and a dominant pattern of intestinal type carcinoma [7]. Furthermore, Shah et al have demonstrated that these GC subtypes, classified on the basis of histology and anatomic location, have also distinct gene expression profiles, supporting the hypothesis that GC subtypes may be distinguished molecularly [8].

One of the clinical implications of this heterogeneity in GC biology is the possible different sensitivity to chemotherapy treatment among the different GC subtypes. Currently, the choice of medical treatment for advanced GC does not take into account the clinical and pathological heterogeneity of this disease. Nevertheless, differences in response to treatment between different subtypes have been reported by a subset analysis of the FLAGS trial, which showed better overall for patients with diffuse GC when treated with cisplatin/S-1 compared to cisplatin/5-FU [9].

Aim of our analysis was to compare the clinical outcome, in terms of response rate, RR, progression-free survival, PFS, and overall survival, OS according to different GC subtypes (1,2,3) in advanced gastric cancer patients receiving first-line platinum-based chemotherapy.

## Patients and Methods

### Patients Selection

The study population was selected from a central database including patients with gastric cancer treated and followed at our institution. Clinical data were retrieved from medical charts. Patients with histologically confirmed, inoperable locally advanced, recurrent or metastatic gastric or gastroesophageal junction adenocarcinoma who have received a first-line combination chemotherapy with a two or three-drugs regimen were included in our analysis. Patients were eligible if they had measurable or non measurable disease; Eastern Cooperative Oncology Group performance status 0–2; age ≥18; no central nervous system metastasis. Patients with HER-2 positive gastric cancer, including patients treated with trastuzumab, were excluded from our analysis.

The patients were divided in three subtypes as previously described:

type 1, proximal non-diffuse GC, with the bulk of the tumour (>80%) located in the gastric cardia, with possible extension to the distal part of the esophagus, and characterized by a non-diffuse pattern of infiltration;
type 2, diffuse GC, located anywhere in the stomach with an entirely diffuse pattern of infiltration with no gland-forming, intestinal type of carcinoma;
type 3, distal non-diffuse GC, with the bulk of the tumour located in the distal or mid part of the stomach and a dominant pattern of intestinal type carcinoma, with or without components of poorly differentiated carcinoma

Gastric cancer subtypes were determined retrospectively reviewing the pathological report for each patient.

### Ethics Statement

This study was approved by Ethical committee AOU Ospedali Riuniti – Umberto I of our institution. All patients provided informed written consent.

### Treatment and Response evaluations

Chemotherapy regimens administered to patients included: fluoropyrimidines and platinum based doublets, as cisplatin+5-fluorouracil (5-FU), cisplatin+capecitabine, oxaliplatin+FU (FOLFOX) or capecitabine+oxaliplatin; 5-FU+irinotecan (FOLFIRI) or 5-FU+mytomicin C combinations; three drugs regimens including anthracyclines (epirubicin, fluoropyrimidines and a platinum derivate combinations as ECF, EOX, PELF), taxanes (docetaxel, fluoropyrimidines and a platinum derivate combinations as TCF or TOX) and mytomicin C (mytomicin C, fluoropyrimidines and a platinum derivate combinations). Physical examination, complete blood counts and biochemical tests were carried out before each cycle of therapy. A chest and abdomen CT scan was performed every three months and when disease progression was clinically suspected by the treating physician to document the extent of disease and to evaluate the response to treatment. The response was assessed using the Response Evaluation Criteria in Solid Tumours (RECIST) 1.0. After the end of treatment, patients were followed every three months with laboratory and imaging studies according to our internal guidelines. Disease status at last follow-up and cause of death were determined by the medical record and death certificates.

### Statistical analysis

Patient, tumour, and treatment variables were compared between the three subgroups using the chi-square for categorical variables. For statistical analysis, overall survival (OS) and progression-free survivial (PFS) were defined, respectively, as the interval between the first day of first-line chemotherapy until the time of the first occurrence of progression, death from any cause or to the date of last follow-up visit and as the interval between the first day of first-line chemotherapy to the date of death or to the date of the last follow-up visit. Kaplan–Meier method was used to estimate PFS and OS curves in the three subgroups; PFS and OS were compared using the log-rank test and we used Cox-regression models for survival multivariate analysis. Tested variables included gender, ECOG PS (0–1 vs 2), stage (locally advanced vs metastatic disease), first-line chemotherapy (three drugs vs two drugs regimen), previous use of adjuvant or neoadjuvant treatment, peritoneal carcinosis, number of metastatic sites (1 vs ≥2), use of second line chemotherapy and disease subtype (1, 2 or 3) as previous defined. We compared the overall response rates (ORR) between the two groups, including complete response and partial response, using the chi-square test.

## Results

### Characteristics of the patients

A total of 248 advanced gastric cancer patients treated with chemotherapy between January 2003 and December 2011 were included in our analysis. Patients characteristics are summarized in Table 2. The majority of patients were males (65.3%); consistently with epidemiological data the proportion of female patients was found to be higher in group 2. The 11.3% of patients (28 patients) presented with proximal non-diffuse GC and were classified as type 1; 112 patients (45.2%) had a diagnose of diffuse GC and were classified as type 2, while type 3 GC, distal-non diffuse GC, included 108 patients (43.5%).

**Table 2:** Patients' Characteristics.

		Type 1	Type 2	Type 3	
		(28 pts)	(112 pts)	(108 pts)	
**Age**	Median (Range),ys	63 (51–76)	61 (29–85)	66 (39–81)	
**Gender**	M	24	60	78	p = 0.0008
	F	4	52	30	
**ECOG PS**	0–1	24	78	84	p = 0.14
	2	4	34	24	
**Surgery**	Yes	14	60	70	p = 0.16
	No	14	52	38	
**Prior Neoadjuvant/Adjuvant Chemotherapy**	Yes	2	28	22	p = 0.11
	No	26	84	86	
**Stage**	Locally advanced	2	10	4	p = 0.28
	Metastatic	26	102	104	
**Metastatic Sites**	1	12	54	62	p = 0.55
	≥2	14	48	42	
**Peritoneal Carcinosis**	Yes	8	56	32	p = 0.004
	No	20	56	76	
**Second Line Chemotherapy**	Yes	18	60	59	p = 0.59
	No	10	52	49	

The 3 groups of patients resulted comparable for most of baseline characteristics of clinical relevance (age, ECOG PS, stage, number of metastatic sites). Most of the patients (91.9%) had metastatic disease while the remaining 8.1% presented with locally advanced, unresectable disease. More than a half of patients (58.1%) had undergone previous surgery for their disease and 20.9% of the patients also received neoadjuvant or adjuvant chemotherapy. As expected, type 2 GC patients, presenting diffuse histology, had a higher incidence of peritoneal carcinosis compared to other groups (50% vs 29.6% in type 3 and 28.6% in type 1).

### Treatment

One-hundred and forty-seven patients (59.3%) received a two drugs fluoropyrimidine-based chemotherapy regimen as first-line treatment. Most of the patients received a combination including a platinum derivate and a fluoropyrimidine (100 patients); the remaining 47 patients received a fluoropyrimidine and irinotecan (22 patients) or mytomicin C (25 patients).

One-hundred and one patients (40.7%) received a three drugs regimen as first-line combination chemotherapy, including a platinum derivate, a fluoropyrimidine and a third drug; in particular, 35 patients received an anthracyclines based combination, 27 patients received a taxanes based regimen while 39 patients received a triplet containing mytomicin. No significant difference was found between the three groups of patients in the use of three or two drugs regimen (p = 0.18). First-line regimens administered in the two groups of patients are summarized in Table 3. A total of 137 patients (55.2%) received a second line chemotherapy, with no significant differences between the three GC subtypes. FOLFIRI (5-fluorouracil, leucovorin and irinotecan) was the most commonly used regimen in this setting. Other second line treatments included taxanes (paclitaxel or docetaxel) and FOLFOX (5-fluorouracil, leucovorin and oxaliplatin).

**Table 3:** First-line chemotherapy regimens administered in the three groups of patients.

	Type 1	Type 2	Type 3
	n. pts (%)	n. pts (%)	n. pts (%)
**Two drugs regimens**	16 (57.1%)	60 (53.6%)	71 (65.7%)
Fluoropyrimidine+platinum derivate	12	40	48
Fluoropyrimidine+irinotecan	4	8	10
Fluoropyrimidine+mytomicin C	0	12	13
**Three drugs regimens**	12 (42.9%)	52 (46.4%)	37 (34.3%)
Anthracyclines-containing chemotherapy	4	18	13
Taxanes-containing chemotherapy	3	14	10
Mytomicin-containing chemotherapy	5	20	14

### Efficacy

**Figure 1:**
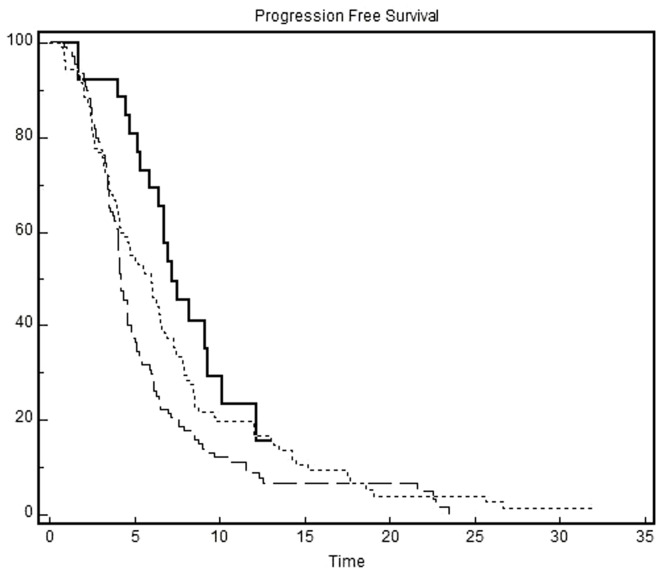
Progression-free survival according to subtypes. ________ type 1 proximal non diffuse GC (median PFS = 7.2 months). ------------ type 2 diffuse GC (median PFS = 4.2 months). ·············type 3 distal non diffuse GC (median PFS = 5.9 months). P = 0.011.

**Figure 2:**
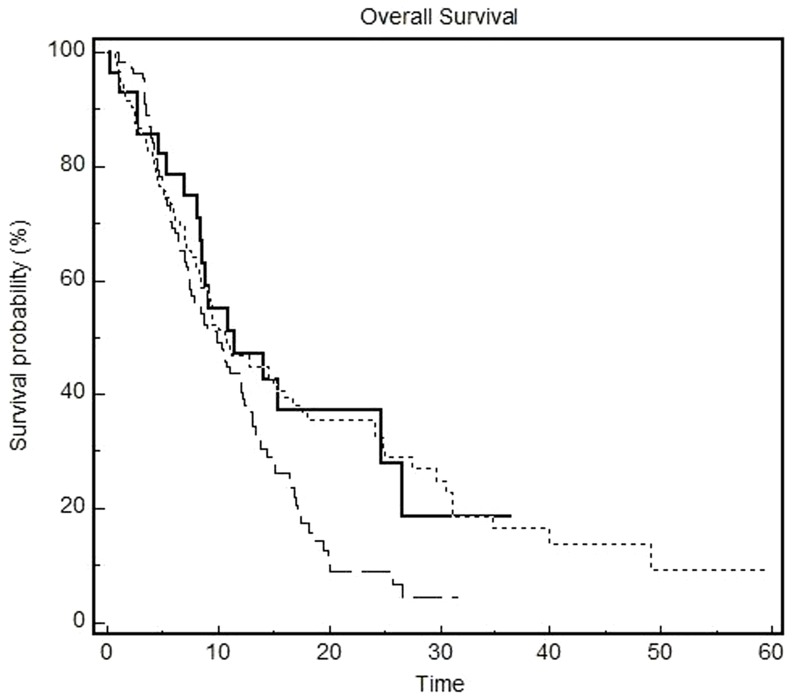
Overall survival according to subtypes. ________ type 1 proximal non diffuse GC (median OS = 11.5 months). ------------ type 2 diffuse GC (median OS = 9.8 months). ·············type 3 distal non diffuse GC (median OS = 11.0 months). P = 0.022.

In 223 patients with measurable disease, the overall response rate to first-line chemotherapy in patients was 29.6%. We observed significant differences in response rate according to disease subtype: the higher response rate was reported in subtype 1 (12/26 patients, 46.1%), while response rate was 34.3% in subtype 3 patients and 20.4% in patients with type 2 gastric cancer (p = 0.015), as described in Table 4. Subtype 2 gastric cancer patients presented a shorter PFS compared to other subgroups with a median PFS of 4.2 months compared to a median PFS of 7.2 months for type 1 patients and 5.9 months for type 3 (p = 0.011) (Figure 1). These differences translated in statistically significant differences in OS. In particular, median OS was 9.8 months in subtype 2 patients compared to a median OS of 11.5 months in subtype 1 and 11.0 months in subtype 3 patients (p = 0.022) (Figure 2). At survival multivariate analysis, when we considered together the two disease subtypes with better outcome (type 1 and type 3), type 2 gastric cancer subtype was found to be an independent predictor for OS (HR = 1.41; 95% CI 1.02–1.94, p = 0.038) together with previous surgery (HR = 0.55; 95% CI 0.39–0.79, p = 0.0012), use of adjuvant or neoadjuvant treatment (HR = 0.57; 95% CI 0.35–0.90, p = 0.018) and use of second line chemotherapy (HR = 0.53; 95% 0.39–0.73, p = 0.0001).

**Table 4:** Response Rate to first-line chemotherapy according to tumour subtype (in 223 patients with measurable disease).

Response Rate	Type 1	Type 2	Type 3
**Overall RR**	12/26 (46.1%)	20/98 (20.4%)	34/99 (34.3%)
**Partial Response**	12/26 (46.1%)	20/98 (20.4%)	30/99 (30.3%)
**Complete Response**	0/26 (0%)	0/98 (0%)	4/99 (4.0%)
**Stable Disease**	9/26 (35.7%)	34/98 (34.7%)	30/99 (30.3%)
**Progressive Disease**	5/26 (18.2%)	44/98 (44.9%)	35/99 (35.4%)

RR: Response Rate.

## Discussion

Advanced gastric cancer is an aggressive disease with high mortality rate. Combinations chemotherapy is the treatment of choice for advanced GC but despite recent progress in cancer treatment, patients' prognosis remains dismal. Currently, even if GC heterogeneity is well recognized, medical management of gastric cancer is not influenced by epidemiological, histological or anatomical considerations. Our analysis has shown how clinical outcome of advanced GC patients treated with chemotherapy is different in terms of RR, PFS and OS according to histology and tumour location, with proximal non-diffuse GC presenting the more favourable outcome.

Clinical implications of GC biological heterogeneity are increasingly being identified in recent trials. In a subset analysis of a phase II trial evaluating bevacizumab with a modified DCF regimen (docetaxel, cisplatin, 5-fluorouracil) in advanced GC patients [10] diffuse GC was shown to have significantly worse PFS and OS compared to other subtypes.

Diffuse tumours also presented the worse response rate with 38% compared to 56% of distal/body diffuse GC and 85% of proximal non-diffuse GC. Interestingly, in this trial gastroesophageal tumours, usually considered more aggressive and presenting a worse prognosis compared to tumours arising from the rest of the stomach [11], [12] had a greater benefit from the treatment. Authors suggested that bevacizumab may be more active in this subset, proximal non-diffuse GC, and may improve the clinical outcome of patients overcoming the adverse prognosis characteristics. Along with these data, our analysis has shown an adverse prognosis for type 2 advanced GC patients treated with combination chemotherapy. Nevertheless, type 1 (proximal non-diffuse) GC patients in our study presented a lower response rate, about 46%, compared to that observed in the phase II trial by Shah et al. A possible explanation is that a more than a half of patients (59.3%) in our study did not receive a three-drugs chemotherapy regimen but a two drug combination and none of the patients received bevacizumab. Suggestions of variable efficacy of treatment on the basis of disease subtype in GC have been observed also in large phase III trial, such as the already cited FLAGS or the ToGA trial. The ToGA trial was an international phase III trial that randomized HER2 positive advanced GC patients to cisplatin and capecitabine/fluorouracil plus trastuzumab or to chemotherapy alone [13]. HER2 expression is more common in intestinal type tumours than in other subtypes [14] and indeed diffuse GC represented only 9% of all patients enrolled in the trial. A subset analysis showed that the addition of trastuzumab to chemotherapy in this subgroup of patients had no effect on survival with a HR for OS of 1.07 (0.56–2.05) versus a HR of 0.69 (0.54–0.88) for intestinal type GC patients.

Evidences of different response to treatment between GC subtypes have been reported not only in patients with advanced disease but also in the adjuvant setting. In particular, in a recent update of INT-0116 study, evaluating post-operative chemo-radiotherapy in patients with resected GC, it has been shown that the benefit of adjuvant treatment is minimal in patients with diffuse histology while is significant in all the other subsets [15]. Similar findings have been observed also in the ITACA-S trial, a multicenter phase III trial comparing 5-fluorouracil and leucovorin versus a sequential regimen including irinotecan and 5-fluorouracil followed by cisplatin and docetaxel in the adjuvant treatment of resected GC patients. In a subgroups analysis of the trial, which could not demonstrate any benefit for the intensive treatment versus the fluorouracil monotherapy, the authors compared the outcome of patients according to disease subtype, dividing the patients in type 1, type 2 and type 3 as defined by Shah et al. The subtype 2, diffuse GC, presented a worse outcome in terms of OS compared to type 3 (HR = 1.35; 95% CI 1.06–1.72, p = 0.016) while no significant difference in terms of prognosis was found between subtype 1 and 3. None of the three subtypes showed a benefit for the experimental arm versus the 5-fluorouracil arm [16].

Although we think that our results are interesting and relevant, we acknowledge that a few points should be more accurately discussed. Firstly, our analysis excluded HER-2 positive GC patients. Even if we believe that also HER-2 positive GC requires a classification, we decided to exclude these patients from our study considering the different biology of this disease, the potential prognostic role of HER-2 expression and the possible confounding factor represented by trastuzumab treatment. The use of different chemotherapy regimens in the study population may affect the results, however we found no difference in the use of two or three drugs regimens between the three subtypes of patients. Moreover, the number of patients in some subgroups, in particular subtype 1 patients, is low and the difference observed in OS, although statistically significant, is actually small and of questionable clinical value. Nevertheless, in our study this difference in OS is associated with a relevant difference in response rate and PFS between the three subgroups of patients. Our analysis suggests that different GC subtypes may present different sensitivity to chemotherapy treatment. Future clinical trials, evaluating chemotherapy treatment in advanced GC patients, should take in account these differences for a better stratification of patients. Indeed, one of the possible explanation for the negative results of recent phase III trials evaluating new cytotoxic or target therapies, such as FLAGS or AVAGAST [17] is that GC is a heterogeneous disease with different biology that may affect response to treatment. Actually, preliminary evidences have shown that the three distinct GC subtypes identified on histopathologic and anatomic criteria, present different gene expression profiles [7]. In the last few years, several studies have demonstrated that molecular markers may correlate to either response or toxicity to specific antineoplastic drugs in GC [18]. Differential expression of biological factors involved in chemotherapy activity, including targets of chemotherapeutic agents, such as thymidylate synthase (TS) for 5-FU, but also genes involved in drug metabolism, may explain the different response to treatment observed in our study. For example, a study by Kamoshida S et al assessed expression of TS, DPD (dihydropyrimidine dehydrogenase) and TP (thymidine phosphorylase) levels in different tumour types, including intestinal-type and diffuse-type gastric adenocarcinoma. The authors found high level of expression of TS, which may be associated with poor response to 5-FU based chemotherapy, in diffuse GC while TS was not overexpressed in intestinal type GC [19].

In conclusions, our study suggests that response to chemotherapy may be different in advanced GC patients, according to tumour histology and anatomic location. Genetic and translational studies are warranted to improve the understanding of molecular drivers and pathways of different GC subtype that may help to identify prognostic and predictive biomarkers as well as to identify specific targets for therapy.

## References

[1] Jemal A, Bray F, Center MM, Ferlay J, Ward E, et al (2011) Global cancer statistics. CA Cancer J Clin 61: 69–90. 21296855 10.3322/caac.20107

[2] Peek RMJ, Blaser MJ (2002) Helicobacter pylori and gastrointestinal tract adenocarcinomas. Nat Rev Cancer 2: 28–37. 11902583 10.1038/nrc703

[3] You WC, Blot WJ, Li JY, Chang YS, Jin ML, et al (1993) Precancerous gastric lesions in a population at high risk of stomach cancer. Cancer Res 53: 1317–21. 8443811

[4] Carneiro F, Huntsman DG, Smyrk TC, Owen DA, Seruca R, et al (2004) Model of the early development of diffuse gastric cancer in E-cadherin mutation carriers and it's implications for patient screening. J Pathol 203: 681–7. 15141383 10.1002/path.1564

[5] Marrelli D, Roviello F, de Manzoni G, Morgagni P, Di Leo A, et al (2002) Different patterns of recurrence in gastric cancer depending on Lauren's histologic type: longitudinal study. World J Surg 26: 1160–5. 12209247 10.1007/s00268-002-6344-2

[6] Liu L, Wang ZW, Ji J, Zhang JN, Yan M, et al (2013) A Cohort Study and Meta-Analysis between Histopathological Classification and Prognosis of Gastric Carcinoma. Anticancer Agents Med Chem 13:2: 227–234. 22934699 10.2174/1871520611313020007

[7] Shah MA, Kelsen DP (2010) Gastric cancer: A primer on the epidemiology and biology of the disease and an overview of the medical management of advanced disease. J Natl Compr Canc Netw 8: 437–47. 20410336 10.6004/jnccn.2010.0033

[8] Shah MA, Khanin R, Tang L, Janjigian YY, Klimstra DS, et al (2011) Molecular Classification of Gastric Cancer: A New Paradigm. Clin Cancer Res 17: 2693–2701. 21430069 10.1158/1078-0432.CCR-10-2203 PMC3100216

[9] Ajani JA, Rodriguez G, Bodoky V, Moiseyenko V, Lichinitser M, et al (2009) Multicenter phase III comparison of cisplatin/S-1 (CS) with cisplatin/5-FU (CF) as first-line therapy in patients with advanced gastric cancer (FLAGS): secondary and subset analyses [abstract]. J Clin Oncol 27 (Suppl 1) Abstract 4511.

[10] Shah MA, Jhawer M, Ilson DH, Lefkowitz RA, Robinson E, et al (2011) Phase II Study of Modified Docetaxel, Cisplatin, and Fluorouracil With Bevacizumab in Patients With Metastatic Gastroesophageal Adenocarcinoma. J Clin Oncol 29 (7) 868–874. 21189380 10.1200/JCO.2010.32.0770 PMC3646322

[11] Sakaguchi T, Watanabe A, Sawada H, Yamada Y, Tatsumi M, et al (1998) Characteristics and clinical outcome of proximal third gastric cancer. J Am Coll Surg 187: 352–357. 9783780 10.1016/s1072-7515(98)00191-4

[12] Kattan MW, Karpeh MS, Mazumdar M, Brennan MF (2003) Postoperative nomogram for disease-specific survival after an R0 resection for gastric carcinoma. J Clin Oncol 21: 3647–3650. 14512396 10.1200/JCO.2003.01.240

[13] Bang YJ, Van Cutsem E, Feyereislova A, Chung HC, Shen L, et al (2010) Trastuzumab in combination with chemotherapy versus chemotherapy alone for treatment of HER2- positive advanced gastric or gastro-oesophageal junction cancer (ToGA): A phase 3, open-label, randomised controlled trial. Lancet 376: 687–697. 20728210 10.1016/S0140-6736(10)61121-X

[14] Tanner M, Hollmen M, Junttila TT, Kapanen AI, Tommola S, et al (2005) Amplification of HER-2 in gastric carcinoma: association with Topoisomerase II-alpha gene amplification, intestinal type, poor prognosis and sensitivity to trastuzumab. Ann Oncol 16: 273–78. 15668283 10.1093/annonc/mdi064

[15] Smalley SR, Benedetti JK, Haller DG, Hundahl SA, Estes NC, et al (2012) Updated analysis of SWOG-directed intergroup study 0116: a phase III trial of adjuvant radiochemotherapy versus observation after curative gastric cancer resection. J Clin Oncol 30 (19) 2327–33 Epub 2012 May 14. 22585691 10.1200/JCO.2011.36.7136 PMC4517071

[16] Bajetta E, Floriani E, Di Bartolomeo M, Labianca R, Falcone A, et al (2012) Itaca-s (intergroup trial of adjuvant chemotherapy in adenocarcinoma of the stomach) trial: comparison of a sequential treatment with irinotecan (cpt-11)+5- fluorouracil (5fu)/folinic acid (lv) followed by docetaxel and cisplatin versus a 5- fu/lvregimen as postoperative treatment for radically resected gastric cancer. Tumori (supp) 13; 3: S1.

[17] Ohtsu A, Shah MA, Van Cutsem E, Rha SY, Sawaki A, et al (2011) Bevacizumab in combination with chemotherapy as first-line therapy in advanced gastric cancer: a randomized, double-blind, placebo-controlled phase III study. J Clin Oncol 29 (30) 3968–76 Epub 2011 Aug. 21844504 10.1200/JCO.2011.36.2236

[18] Scartozzi M, Bittoni A, Pistelli M, Galizia E, Berardi R, et al (2009) Toward molecularly selected chemotherapy for advanced gastric cancer: state of the art and future perspectives. Cancer Treat Rev 35 (5) 451–62 Epub 2009 May 20. 19467788 10.1016/j.ctrv.2009.04.008

[19] Kamoshida S, Shiogama K, Shimomura R, Inada K, Sakurai Y, et al (2005) Immunohistochemical demonstration of fluoropyrimidine-metabolizing enzymes in various types of cancer. Oncol Rep 14 (5) 1223–30. 16211289

